# Viscosity study of maternal and formula milks according to the assessment proposed by the International Dysphagia Diet Standardisation Initiative Model: a comparison with Rheological Assessment

**DOI:** 10.1590/2317-1782/e20240049en

**Published:** 2025-02-24

**Authors:** Ana Maria Hernandez, Maria Isabel Berto, Esther Mandelbaum Gonçalves Bianchini

**Affiliations:** 1 Programa de Estudos Pós-graduados em Fonoaudiologia, Pontifícia Universidade de São Paulo - São Paulo (SP), Brasil.; 2 Instituto de Tecnologia de Alimentos, Centro de Tecnologia de Laticínios - Campinas (SP), Brasil.

**Keywords:** Dysphagia, Fluoroscopy, Viscosity, Formula, Human Milk

## Abstract

**Purpose:**

The present study aimed to investigate the behavior and the viscosity classification of liquids recommended for infants, up to six months, in Brazil, and their formulations used in Videofluoroscopy Swallowing Studies.

**Methods:**

Breast milk from different gestational and breastfeeding time, two regular infant formulas, and one anti-reflux formula were evaluated in five different formulations: pure, mixed with liquid barium sulfate, in two proportions (20 and 33%), thickened with a specific thickener for neonates and thickened and mixed with 20% liquid barium sulfate, using the International Dysphagia Diet Standardisation Initiative model. The results were compared with data obtained from a previous rheological study.

**Results:**

The breast milk samples and the infant formulas, in pure presentation, exhibited similar behavior except for the anti-reflux formula. The thickened samples with the addition of barium and the pure liquid barium sulfate exhibited the highest viscosity among the formulations. The increase in viscosity with the addition of barium occurred for all samples and for the thickened formulations, but not in linear behavior. The results showed minor differences of IDDSI classification compared to the findings obtained in the rheological study.

**Conclusion:**

The present study revealed the variability of liquid viscosity across different formulations, supporting the idea of the importance of this knowledge in videofluoroscopic assessment. It has also highlighted the risk associated with using a subjective method in preparing the stimuli offered, allowing for greater reliability in diagnosing swallowing dynamics in neonates and infants. This approach may help prevent inaccurate diagnoses and harmful interventions.

## INTRODUCTION 

Videofluoroscopy swallow studies (VFSS) are commonly regarded as the 'gold standard' technique to assess dysphagia. The accuracy of dysphagia diagnosis in pediatric VFSS depends on several variables that can influence the swallowing dynamics, as well as on the confidence that the products used during the radiological procedures are representative of the typical liquids that infants fed on^(1,2)^. During a VFSS, thickeners should be added to assess the safe consistency for the patient and the stimulus must be impregnated with radiopaque material to visualize the bolus radiographically during swallowing. Studies have been conducted with the aim of understanding the behavior of the products commonly used in the care of dysphagic patients, and their impact on the swallowing dynamics^(2,3)^. Specifically, considering the VFSS of infants up to six months, it is crucial to understand the behavior of breast milk and infant formulas, when a thickener and the radiopaque material, usually barium sulfate, are added, given that liquids are their exclusive sources of nutrition and hydration^(4-7)^. Understanding this behavior is of great importance, especially in countries and institutions which lack access to pre-prepared products, in a range of consistencies from thin to moderately thick, available on the market. Consequently, clinicians must prepare their own VFSS stimuli, by mixing barium and thickener with regular liquids, in a subjective manner, classifying the consistency by observing the flow from a spoon^(6,7)^. In addition, it is imperative that the liquids tested are representative of those used in the infant’s routine diet, aiming to reproduce flavors, textures and viscosity as closely as possible. This ensures better cooperation during the evaluation and greater precision in recommending a safe diet for the patient, by replicating the levels of viscosity and thickness observed during the VFSS^(1,6,7)^. Therefore, it is fundamental to know the behavior of liquids in various formulations, exercising caution when preparing the stimulus to be offered during the VFSS.

Rheology can contribute to this knowledge, since it objectively analyzes properties of foods such as viscosity and shear rate, by characterizing food behavior in complex deformations, such as those encountered during swallowing^(8,9)^. In the pursuit of better understanding of how liquids behave, researchers used mainly rheological methods as their first choice^(10,11)^. Considering the typical diet of infants, specifically breast milk, there is a notable lack of research. Prior studies have suggested that liquids used in the test situation were not representative of typical liquids indicated for infants^(1,5)^. Some studies have highlighted the impact of time on formulations with thickeners^(4,12,13)^, while others have focused on the differences in the amount of barium added to infant formulas^(14)^. To date, there have been few studies that have evaluated breast milk and infant formulas in their various formulations. A rheological study, which evaluated breast milk, regular and anti-reflux infant formulas, as well as different forms of barium sulfate, concluded that the addition of barium to any liquid increases its viscosity, albeit in different ways^(6)^. It is important to note that all liquids were evaluated only with the addition of powdered barium sulfate, diluted in water. Additionally, the products evaluated were specific to the country of origin of the study and did not include any formulations containing thickener. The assessment of the two breast milk samples demonstrated performance variability, underscoring the need for further research^(6)^.

The concern regarding these aspects, primarily in the approach with infants up to six months of age, prompted another rheological study to analyzetwo types of breast milk and Brazilian typical infant formulas, commonly recommended for this population. A notable distinction in this research was the use of a thickener specifically indicated for neonates and for breast milk. The study evaluated these liquids in their pure form as well as in various formulations, highlighting their variability and level of viscosity^(7,15)^.

Despite the effectiveness of the Rheology and its significant contribution to this field of knowledge, it remains an expensive method and is not readily accessible to clinicians and caregivers, as it requires specific equipment and specialized professionals^(8,9,15)^.

Pursuing the same purpose as a consensual guideline, the *International Dysphagia Diet Standardisation Initiative (IDDSI)* proposed a flow test, a terminology and definitions for food and liquid textures applicable to all individuals and ages^(16)^. This proposal provides an accessible parameter in the preparation and handling of liquids to be offered in dysphagic individuals’ routine diets^(16-18)^. Therefore, the model proposed by IDDSI can be used to analyze breast milk and first infant formulas in different samples, allowing for the comparison of samples from multiple donors since the volume required for analysis is smaller than that in the rheological evaluation.

Comparing different techniques and methods for measuring food viscosity can enhance reproducibility and quality control in liquid thickening. This ensures that the thickness level is as expected and safe for patients with dysphagia^(19, 20)^_._ The considerations above justify the exploratory study evaluating viscosity of liquids, regularly recommended in Brazil, offered in pediatric VFSS, comparing the data obtained through rheological versus IDDSI flow test.

The current research aimed to investigate the behavior of standard formulas recommended in Brazil for infants up to six months, and their similarity to the viscosity of several samples of breast milk, in various formulations, including a thickener specific for newborn. It aimed to compare and discuss objective viscosity measurements considering both practical measurements (IDDSI) and fundamental rheological analysis.

## METHODS

This study followed the guidelines and regulatory standards of Resolution 196/96 of the Ministry of Health’s National Health Council (2013) and was approved by the Ethics Committee under certificate number 63361616.2.0000.54. Participation in the research was voluntary and took place only after the donor mothers agreed to participate and signed an informed consent form specific to the study.

All samples were evaluated according to the IDDSI model, which involves measuring the remaining volume of 10 ml of the liquid after it flows freely for 10 seconds in a 10 ml syringe. Based on the residual volume, the sample is characterized in terms of its fluidity, using numerical categories, from zero to four, corresponding to the consistency of the liquid, from thin to extremely thick^(16-18)^.

At the first stage, nine samples of fresh breast milk and four samples of pasteurized breast milk were evaluated, in pure formulation and with the addition of 33% liquids barium sulfate Bariogel^®^, comparing their performance and their possible viscosity difference according to gestation time and breastfeeding time.

The subsequent phase entailed a comparative analysis of two fresh breast milk samples, two standard infant formulas, and an anti-reflux formula. The infant formulas and radiopaque product used for this study were selected based on frequency of use in Brazilian settings. All of these options were recommended for infants up to six months of age. They underwent analysis in their pure form, as well as with two different proportions of added liquid barium sulfate Bariogel^®^ (20% and 33%), while the other formulations included the addition of a measure of thickener labeled Aptamil^®^ Feed Thickener with one of them also containing 20% barium (Bariogel^®^). For each formula and thickener combination, initially, each sample was prepared according to the instructions using the scoop or recommendations provided by the manufacturer. After the products had been evaluated in the different forms of preparation, the data were compared with the findings of the rheological evaluation^(7,15)^.

The classification of liquids, according to the viscosity and thickening criterion of both methods used in the study, proposes a specific extension range, with a minimum threshold and a maximum threshold for each category. That is, category 1 in the IDDSI methodology, classified as slightly thick, has a minimum residue threshold of 1 ml in the syringe, while the maximum threshold would be 3.9 ml.

In the current research each category was divided into three thresholds. The initial 33% was the low threshold, the intermediate 33% was the medium threshold, and the last 33% was the high threshold. Specifically, in the rheological classification from 1 to 50 cP,*^^1^^ the low threshold ranged from 1 to 16.4 cP; the medium threshold, from 16.5 cP to 33 cP; and the high threshold, from 33.1 cP to 50 cP. The same method was used in all categories.

## MATERIAL

### Breast milk

First stage: Nine samples of fresh milk and four of pasteurized milk were collected, in the hospital, from mothers of infants aged up to six months, with varying lengths of gestation (26 4/7 to 38 5/7 weeks, mean 34 1/7 weeks, standard deviation 4 3/7 weeks) and breastfeeding (from 2 to 21 weeks, mean 9 4/7 weeks, standard deviation 1 3/7 weeks).

Second stage: Two samples of fresh breast milk from the same time of gestation (39 weeks) and lactation (12 weeks) were evaluated in various formulation modes: pure, added with a measure (1.7 g) of a specific thickener for breast milk, labeled as Aptamill^®^ Feed Thickener produced by Nutricia, another sample of the liquid added one measure of the same thickener plus 20% of barium sulfate. This thickener is a product indicated for newborns and infants over 42 weeks of corrected gestational age (CGA).

### Infant Formulas

Formulas tested are listed in Chart 1. The flow of infant formula was evaluated pure, with the addition of liquid barium sulfate, Bariogel^®^, in the proportions of 20% and 33%. The same samples were evaluated in formulation with thickener Aptamil^®^ Feed Thickener and in formulations with the same thickener plus addition of 20% liquid barium sulfate (Bariogel^®^).

**Chart 1:** Products used in testing

Type	Registration owner	Brand name
Standard formulas	Nutricia	Aptamil® Pro 1
	Mead Johnson	Enfamil® Premium 1
Specialized Formula	Mead Johnson	Enfamil® A.R
Thickener	Nutricia	Aptamil® Feed Thickener
Radiopaque material	Cristália	Bariogel®

### Procedure

Chart 2 describes the preparation of all samples.

**Chart 2:** Preparation of the samples

Formulations	Breast milk	Infant formulas
Pure	The samples were thawed by heating to 40° in a container in a water bath. After that the sample was allowed to cool down to 25 °C for the test.	3 measures of formula (13.4 g) for 90 mL of water, 25 °C for each sample. Mixed as instructed by the manufacturer.
Added 20% barium sulfate Bariogel®	For each 30 mL of breast milk, thawed as described, was added 6 mL of barium. The product was homogenized by pumping with a syringe for 60’ and allowed to cool down to 25 °C for the test.	For each 30 mLof the prepared formula was added 6 mL of barium. The product was homogenized by pumping with a syringe for 60’. Waiting more 60” for the test.
Added 33% barium sulfate Bariogel®	For each 30 mL of breast milk thawed as described, 9.9 ml of barium was added. The product was mixed by pumping with a syringe for 60” and allowed to cool down to 25 °C for the test.	For each 30 mLof the prepared formula was added 9.9 mL of barium. The product was homogenized by pumping with a syringe for 60’. Waiting more 60” for the test.
Added Aptamil® FeedThickener	For each 45 mL of breast milk thawed was added ½ measure of the thickener (0.85 g) mixed at 40 °C, mixed by pumping with a syringe for 60’and was allowed to cool down to 25 °C for the test.	For each 45 mL of prepared formula, was added ½ measure of the thickener (0.85 g). The product was homogenized by pumping with a syringe for 60”. Waiting more 60” for the test.
Added Aptamil® FeedThickener	For each 45 mL of breast milk, thawed, was added ½ measure of the thickener (0.85 g) mixed at 40 °C, homogenized by pumping with a syringe for 60” and 9 mL of barium. The product was mixed by pumping with a syringe for 60”.	For each 45 mL of the prepared formula was added ½ measure of the thickener (0.85 g). and 9 mL of barium.The product was homogenized by pumping with a syringe for 60”. Waiting more 60” for the test.
Plus 20% barium sulfate Bariogel®		

#### Breast milk preparation

The breast milk material was collected, stored, and thawed following the norms of the Breastfeeding Scientific Department of the Brazilian Ministry of Health’s National Society for Pediatric Health^(21)^. Fresh breast milk was collected and pasteurized under the responsibility of the milk bank of two hospitals in São Paulo that admits people belonging to classes C and D, following their protocol.

The samples were tested with two different proportions of added liquid barium Bariogel^®^ (20% and 33%). As recommended by the manufacturer, the measure of the Aptamil^®^ Feed Thickener was added to the milk at a temperature of 40 °C. All the samples were completely diluted by pumping with a syringe for 60 seconds and allowed to cool down to 25 °C for the test. The temperature was controlled with a food thermometer manufactured by Clink^®^, and the liquid flow was verified with 10ml syringes manufactured by Becton and Dickinson^®^ (BD)^(22)^. The syringes were replaced for each sample.

#### Formula preparation

All infant formulas were prepared following the manufacturers’ instructions: three measures (13.4 g) of each formula were diluted in 90 ml of water. The powder was mixed for 60 seconds until complete dilution and another 60 seconds were counted for total hydration, as suggested in a previous study^(15)^. All infant formulas were tested with two different proportions of added liquid barium sulfate, Bariogel^®^ (20% and 33%). After adding the radiopaque material (Bariogel^®)^, the mixture was homogenized by pumping it into a syringe for 60 seconds and another 60 seconds more for total hydration. The same products were tested with the addition of Aptamil^®^ Feed Thickener and another sample with the same thickener with the addition of 20% barium sulfate Bariogel^®^. The formulas were thickened using the same pattern as breast milk.

The procedure followed the IDDSI proposal and consisted of placing 10 ml of milk into the Becton and Dickinson^®^ (BD 303134) syringe placed in a vertical position, with its lower distal tip occluded by a finger. Once the timer was turned on, the tip of the syringe was opened, allowing the liquid to flow for 10 seconds, when it was stopped. Then, the volume of liquid remaining in the syringe was measured^(16,22)^. The procedure was repeated to endorse the result. If there was a discrepancy, a third sample was tested.

The decision to evaluate milk flow at a 20% concentration was influenced by previous studies that performed assessments with lower proportions of barium sulfate, with a good prospect of visualization in VFSS examination^(14,23)^. Furthermore, having access to a thickener suitable for breast milk, which is approved and considered safe for use in newborns, enabled the inclusion of this test in the study^(24)^.

## RESULTS

### Breast milk

Table 1 shows the viscosity measurements (mean, standard deviation) for all breast milk samples, both fresh and pasteurized, in relationship with gestational and breastfeeding times. The pure samples and the samples added with 20% liquid barium sulfate (Bariogel^®^ blended) revealed a predominance of the same low viscosity thresholds. An exception was observed in a colostrum sample from the mother of a seven-day-old newborn (during the first week of breastfeeding), which exhibited a slight increase in viscosity, resulting in a residue of 1.5 ml (11.11%) classified as slightly thick (level 1). Upon adding 33% of liquid barium sulfate Bariogel^®^, the samples showed residues of 3 ml (32.2%) and 4 ml (77.8%), exhibiting slight variability in their behavior. These values represent the highest limit of slightly thick liquid (level 1) or a transition toward mildly thick liquid (level 2) according to the IDDSI classification. Notably, this viscosity increase was approximately 13.5 times greater than that observed in pure milk samples.

**Table 1:** Viscosity measurements of breast milk samples both pure and added liquid barium sulfate Bariogel® in milimetrical residue and in IDDSI category versus gestation time and breastfeeding time

Breast milk pure	Gestation Weeks	Breast feeding Weeks	Residue in mL	IDDSI cate gory	Residue in mL+ 20% Bariogel®	IDDSI cate gory	Residue in mL + 33% Bariogel®	IDDSI cate gory
Sample 1	38	18	1	0			4	2
Sample 2	34	1	1.5	1			4	2
Sample 3	38 5/7	11 2/7	0	0			3	1 HT
Sample 4	26 4/7	3	0	0	0.5	0	3	1 HT
Sample 5	39 2/7	3 1/7	0	0	0.5	0	4	2
Sample 6	29 1/7	14	0	0			4	2
Sample 7	28 2/7	6 6/7	0	0			4	2
Sample 8	37	21 6/7	0	0			4	2
Sample 9	36	2 3/7	0				4	2
Mean	34 1/7	9	0.277		0.5		3.78	
SD	+/- 4 3/7	+/-7 4/7					+/- 0.39	
Pasteurized breast milk pure								
Sample 1	40	23	0	0	0.2	0	3.5	1 HT
Sample 2	41	1	0	0	0.2	0	3.5	1 HT
Sample 3	39	12 6/7	0	0	0	0	4	2
Sample 4	40	6 5/7	0	0	0.1	0	4	2
Mean	40	11 4/7	0	0	0.2	0	3.75	
SD	0.632	7 2/7					0.447	

Caption: HT = high threshold; SD = standard deviation

**Figure 1:**
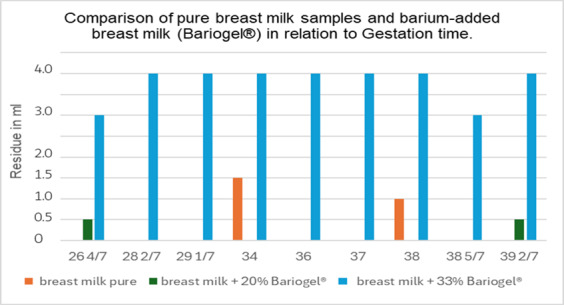
Comparison of pure breast milk samples and barium-added breast milk (Bariogel®) in relation to Gestation time.

**Figure 2:**
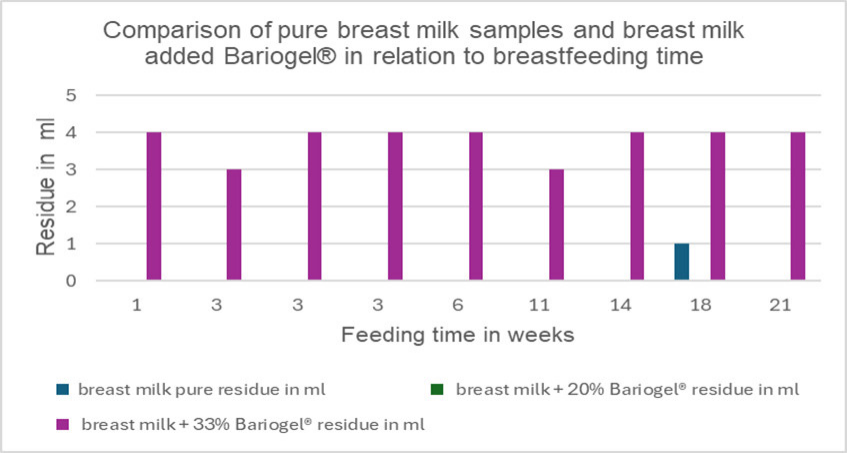
Comparison of pure breast milk samples and breast milk added Bariogel® in relation to breastfeeding time

There was a lack of significant correlation between flow threshold and gestation time, both for the pure product and when 33% barium sulfate is added. Additionally, there was no significant correlation when comparing breastfeeding time at the moment of milk collection. The relevant data can be found in Table 1 and Figures 1 and 2.

The pasteurized breast milk samples exhibited more homogeneous patterns, maintaining 100% similarity in pure milk residues, at the thin liquid threshold (according to the IDDSI classification), with a flow closely resembling that of water. The samples with 33% barium sulfate, increased to 3.5 ml (50%) and 4 ml (50%), residual portion, reaching the higher threshold of a slightly thick liquid, or transitioning towards mildly thick liquid, similar to fresh breast milk samples behavior. (Table 1)

**Figure 3:**
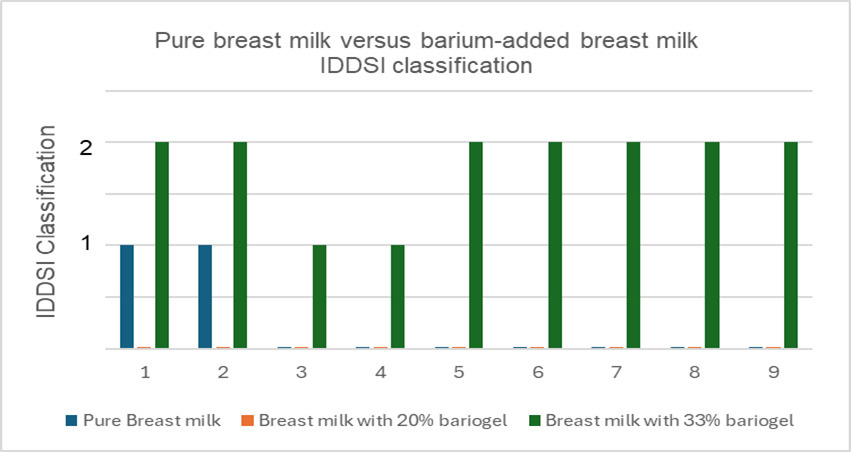
Breast milk pure versus breast milk added barium sulfate Bariogel® - IDDSI classification

Table 1 shows the comparison of performances between pasteurized and fresh breast milk samples revealed slight variability. Applying the IDDSI classification criteria, pure breast milk and pasteurized milk predominantly achieve level 0, as well as with addition of 20% of barium sulfate (Bariogel^®^). When 33% barium sulfate (Bariogel® brand) was added, there was a shift to level 2 at its lowest threshold (77.8%), indicating a mildly thick liquid (see Table 1 and Figure 3).

### Infant formulas

**Figure 4:**
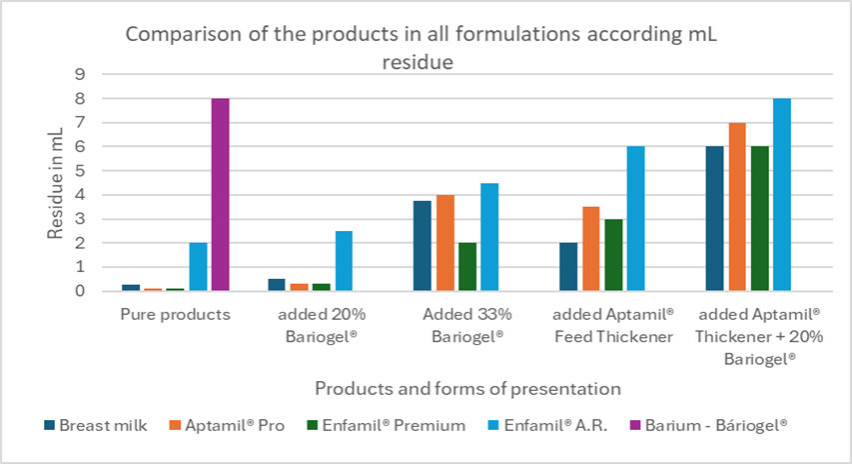
Comparison of all products in all formulations according to ml residue

The performance of samples of Aptamil^®^ Pro and Enfamil^®^ Premium first infant formulas was similar to the mean performance of fresh and pasteurized breast milk, as well as the products added with 20% of barium sulfate (Bariogel^®^). They remained at a thin liquid threshold. Enfamil^®^ A.R. and Bariogel® barium sulfate had a slower flow, respectively classified as slightly thick liquid (level 1) mildly thick to moderately thick (threshold from level 2 to 3). (Table 2, Figure 4)

**Table 2:** Comparison of products in all tested presentation forms. Residue measurements in millimeters and in IDDSI categories

	Pure product		+ 20% barium		+ 33% barium		+ thickener		+ thickener + 20% barium	
	mL	Category	mL	**Cate gory**	mL	Cate gory	mL	Category	mL	Category
Breast milk	0.2	0	0.5	0	3.8	1 HT	*2*	1 LT	6	2
Aptamil® Pro	0.1	0	0.3	0	4	1 -2	3.5	1	7	2
Enfamil® Premium	0.1	0	0.3	0	2	1	3	1	6	2
Enfamil® A.R.	2	1	2.5	1	4.5	2	6	2	8	3 LT
Bariogel®	8	3 LT								

Caption: HT = High threshold; LT = low threshold

The samples with added 33% barium sulfate had an increase in viscosity, varying between the medium and higher limits of level 1, still considered slightly thick liquids (2 mL) or in low threshold to mildly thick (4 mL and 4,5) in the IDDSI classification.

**Figure 5:**
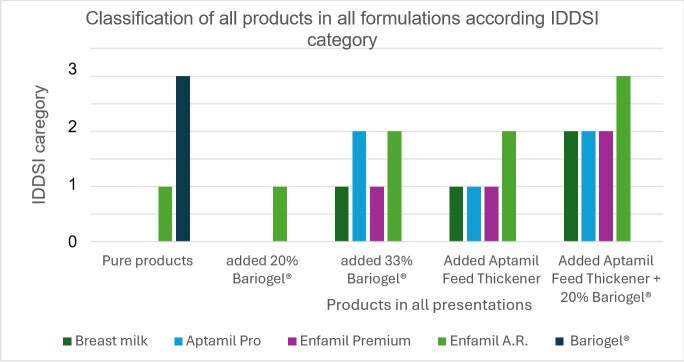
Classification of all products in all formulations according IDDSI category

All the samples exhibited lower viscosity rates than barium sulfate (branded as Bariogel^®^) in its original undiluted formula, except for Enfamil® A.R when thickened with a measure of the thickener Aptamil^®^ Feed Thickener and blended with 20% liquid barium (Table 3, Figures 4 and 5). The viscosity of liquids thickened with one measure of Aptamil® Feed Thickener (1.7 g) increased variably, without a single pattern, varying from an increase of two or three times higher than the pure product.The data are consistent with findings from the previous rheological study ^(15)^ (Table 3, Figure 4).

**Table 3:** Rheological behavior of samples and respective classifications according to NDD and IDDSI and the same samples evaluated according IDDSI proposal and respective classifications. Comparing consistency nominations

Sample	Viscosity at 50 s^-1^ (mPa.s)	Classification based on rheological data - NDD	IDDSI Classification based on rheological	Residue in mL	IDDSI Classification Based on the flow test
Breast milk media	1.70 ± 0.10	Thin LT	Slightly thick *	0.2	Thin
Breast milk + 20% barium - Bariogel®	9.77 ± 0.21	Thin LT	Slightly thick LT *	0.5	Thin MT
Breast milk + 33% barium - Bariogel®	31.30 ± 0.32	Thin MT	Slightly thick MT *	3.78	Slightly thick HT *
Breast milk + 1 measure of thickener	164.79 ±8.84	Nectar thick MT	Mildly thin LT §	2	Slightly thick MT *
Breast milk + Thickener + 20% barium - Bariogel®	305.84 ± 4.24	Nectar thick HT	Mildly thick LT §	6	Mildly thick MT §
Aptamil® Pro 1	1.53 ± 0.06	Thin LT	Slightly thick LT *	0.1	Thin LT
Aptamil® Pro 1 + 20% Bariogel®	7.40 ± 0.10	Thin LT	Slightly thick LT *	0.3	Thin LT
Aptamil® Pro 1 +33% barium - Bariogel®	28.38 ± 0.93	Thin *	Slightly thick MT *	4	Mildly thick LT §
Aptamil® Pro 1 + Thickener	117.07 ± 5.97	Nectar thick §	Mildly thick MT §	3.5	Slightly thick HT *
Aptamil® Pro 1 + Thickener + 20% barium - Bariogel®	257.24 ± 4.85	Nectar thick HT §	Mildly thick HT §	7	Mildly thick HT §
Enfamil® Premium 1	2.40 ± 0.52	Thin LT	Slightly thick LT *	0.1	Thin LT
Enfamil® Premium 1 + 20% Bariogel®	9.23 ± 0.15	Thin LT	Slightly thick LT *	0.3	Thin LT
Enfamil® Premium 1 plus 33% Bariogel®	19.8 ± 0.10	Thin LT	Slightly thick MT *	2	Slightly thick MT *
Enfamil® Premium A.R.	62.08 ± 3.86	Nectar thick LT §	Mildly thick LT §	2	Slightly thick MT *
Enfamil® Premium A.R. + 20% Bariogel®	109.21 ± 13.33	Nectar thick LT §	Mildly thick MT §	2.5	Slightly thick MT *
Enfamil® Premium A.R + 33% Bariogel®	126.16 ± 6.84	Nectar thick LT §	Mildly thick LT §	4.5	Mildly thick LT §
Enfamil® Premium A.R. + Thickener	Evaluation not performed			6	Mildly thick MT §
Enfamil® Premium A.R. + Thickener+ 20% Bariogel®	Evaluation not performed			8	Moderately thick LT +
Sulfato de barium Bariogel®	609.62 ± 94.29	Honey thick +	Moderately thick LT +	8	Moderately thick LT +

millipascal-second (mPa.s) is a unit of dynamic viscosity

* highlight all products in category Slightly thick

§ highlight all products in category Mildly thick

+ highlight all products in category Moderately thick

Caption: HT = High threshold; MT = Medium threshold; LT = low threshold

Table 3 illustrates the correlation between the IDDSI classification of values obtained in our current study and their corresponding measurements in millipoise (mPa.s) from the rheological study. We compare similarities and differences in viscosity naming, as well as divergence in viscosity analysis of the product. (Figures 4 and 5). The present study points to a discrepancy between the IDDSI classifications and the distinctive viscosity thresholds in mPa.s. Out of the fifteen comparable samples (the Enfamil^®^ A.R. formulations with Aptamil^®^ Feed Thickener were not evaluated in the rheological study), only five showed similarity in naming according to the IDDSI classification proposal, based on rheological data and residue data in milliliters. There was no linearity in the discrepancies, just as in the comparison of product naming according to the National Dysphagia Diet (NDD)^(25)^, used in rheological study model, versus the IDDSI model (Table 4).

**Table 4:** NDD and IDDSI Classification of Liquids

NDD		IDDSI	
Centipoise	Classification	Fluid remain	Classification
0-50 cp	Thin	0	Thin
		up to 4 ml	1 – Slightly thick
51-350 cP	Nectar thick	4 to 8 mL	2 – Mildly thick
351-1750 cP	Honey thick	8 to 10 mL	3 - Moderately thick
>1751 cP	Spoon thick	>10 mL	4 – Extremely thick

Caption: Cp (centipoise) is a unit of dynamic viscosity, commonly used in the field of fluid mechanics and rheology. It measures how resistant a fluid is to flow under an applied force or stress

## DISCUSSION

An unexpected and important result observed in the present research was that there was no significant variability in viscosity in the pure breast milk samples analyzed, despite the specialized bibliography pointing to great variability in the composition of breast milk in terms of its composition, influenced by factors such as gestation time, breastfeeding time, health conditions, genotype, and maternal diet^( 26,27)^. These studies revealed that the average amount of protein content gradually decreases from the second to the sixth month of breastfeeding when it stabilizes^(26,27)^. The fat component increases over time, occurring in greater proportion at the end of breastfeeding. The fat fraction is more sensitive to the maternal diet, with less impact on protein and carbohydrates^(27)^. In the present research, the comparison between nine samples of fresh breast milk and four samples of pasteurized breast milk revealed little variability in the flow index, particularly upon the addition of 33% liquid barium sulfate Bariogel^®^, despite the considerable range of breastfeeding time. These results agree with a previous study that analyzed the rheology of breast milk samples from two donors^(15)^. On the other hand, this finding diverges from other rheological research^(6)^, which analyzed two breast milk samples and highlighted variability in their behavior. It is noteworthy that the milk samples in their study were thawed for 24 hours in the refrigerator, unlike the procedure employed in the present study. Performing tests immediately after milk pumping may indicate differences, making it a possible topic for future studies.

The impact of gestational time (GA - gestational age of the newborn at birth) and breastfeeding time, on the date of collection, were assessed as possibly interfering with the viscosity of the milk. This data was not relevant to the present study. This may be justified by possible differences in the components of the milk, according to the breastfeeding time, time of day, and maternal diet conditions – which, however, did not interfere with its viscosity. Future studies should address statistical analysis with controlled variables, such as the time of day when the sample is collected.

The decision to evaluate milk flow at 20% concentration of Bariogel® was interesting due to the fact that a significant addition of radiopaque material could lead to substantial flavor changes in the stimulus, which can make it difficult for the patient to cooperate during the exam. Clinicians dealing with dysphagic patients face the challenge of balancing safe feeding practices with minimal interference in the patient’s dietary preferences. Furthermore, the possibility of using a thickener recommended for breast milk, without usage restrictions for neonates from 42 weeks of gestational age, was an important piece of data in the research. The results obtained from thickening samples of breast milk and Aptamil^®^ Pro infant formula with a measure of Aptamil^®^ Feed Thickener are similar to the findings of a recent study that aimed to establish a recipe for Level 1, (slightly thickened) formula for Australian infant formulas/thickeners. The study achieved Level of slightly thick liquid with one measure of the same thickener, without radiopaque addition, that was not tested^(28)^.

Considering the results of both the evaluation methods and the same samples, the addition of the same thickener, in formulation with 20% of liquid barium sulfate Bariogel® reclassified the liquid to mildly thick, two categories above the consistency of the pure product and just one above the thickened product without the radiopaque material (Bariogel^®^).

Using the IDDSI classification criteria for rheological data, all samples in pure formulation, with the addition of 20% and 33% barium sulfate, would be considered slightly thick liquids, all marked with an asterisk (*) on Table 3, except for the anti-reflux formula. The samples thickened with one measure of Aptamil® Feed Thickener, whether mixed with 20% barium (Bariogel^®^) or not, would match the Aptamil® A.R. product in its three formulations: pure, with 20%, and 33% liquid barium sulfate Bariogel^®^, all marked with the section sign (§) on Table 3. The data should be considered when performing the VFSS.

This observation led us to question the safety of recommending a diet for dysphagic patients based solely on the consistency label, despite it being grounded in specific protocols. When dealing with fragile patients, such as infants at risk of dysphagia, the concern extends beyond the mere nomination of liquids consistency. The application of the same viscosity classification to liquids in their pure form, along with the addition of 20% or 33%, without considering the variability of numerical data (whether in mPa·s rheological measurements or mL residual volume), may not ensure safety in dietary recommendations. It is worth questioning how these small differences, from 1 to 50 mPa.s or from 1mL to 4 mL residual, might impact the swallowing dynamics of neonates or infants, potentially affecting dietary recommendations based solely on categorical labels without specifying numerical values.

In general terms, 75% of the fifteen samples diverged in the viscosity classification, tested with two different nomination and methodology proposals: dynamic viscosity assessment, which is part of the fundamental technique, and kinematic viscosity (IDDSI), an empirical technique. The classification encompasses a range of values, whether in mL of residue in ten seconds or millipascal seconds when evaluated with a rheometer. However, these variations can make a difference in diagnosis and clinical management.

The diversions in the products nomination according the findings in mPa·s evaluation and therefore classified differently, comparing to the category of the classification according NDD versus IDDSI classification (3^nd^ and 4^th^ columns of table 3) can be partially explained by the fact that the IDDSI classification criteria designate as thin liquids only those whose fluidity matches that of water, (specifically below 1 mPa·s). In contrast, the classification proposed by NDD considers thin liquids to be within a range of 1 to 50 mPa·s. Liquids with viscosities between 1 and 50 mPa·s, according to the IDDSI classification, are categorized as slightly thick. (Table 4) When comparing data from the rheological study in NDD classification and the IDDSI criteria, subtle discrepancies become apparent. This minor discrepancy was observed across almost all the samples analyzed, prepared in the same manner, at the specified temperature of 25 °C.

With the IDDSI proposal, progress was made related to the development of a less subjective fluid consistency classification, based on an analytical procedure. However, there is still the possibility for improvements regarding the criteria and ranges used for each designation. Comparing both methodologies, there is a great non-linearity and disproportionality in the viscosity and remaining volume ranges of the NDD and IDDSI classifications, respectively. For lower viscosity liquids classified as Thin and Slightly thick, for example, the NDD classification includes a narrow viscosity range of 50 cP, while the IDDSI remaining volume is up to 4 mL. In contrast, a fluid classified as moderately thick can have viscosity differences of 1399 cP (from 351 to 1750 cP) and only 2 mL changes of remaining volume (8 to 10 mL). This fact may also be another reason to justify the non-agreement in the sample classification according to both methodologies.

A possible proposal to ensure greater accuracy and safety in the preparation of stimuli to be offered in VFSS, especially for neonates and infants up to six months, would be the criterion of three subdivisions in each category, such as low, medium, and high threshold. These considerations can significantly affect diagnosis and clinical decision-making.

Considering the aforementioned data, the variability in the designation of product consistency as well as in the behavior of liquids formulated with barium sulfate and thickener, although small, it is possible to reaffirm and emphasize the risk of using subjective methods to assess the consistency and viscosity of the stimuli in VFSS. and the importance of understanding the behavior of the products used in a known and reproducible protocol.

These findings indicate the importance of a single judicious standard in the preparation of the diet offered in VFSS and in the indication of diets. The proposed IDDSI methodology is an accessible, practical method which can be reproduced by caregivers and professionals responsible for instrumental swallowing examinations.

These considerations are significant for formulating a VFSS exam with appropriate, standardized, and replicable stimuli preparation conditions and techniques, contributing to accurate and effective VFSS diagnosis and the indication of therapeutic procedures. Beyond understanding liquid behavior, the findings of this study allowed us to address the issue related to the boundaries between consistency classification ranges and propose potential ways to make the viscosity classification in VFSS reports even clearer and more specific. In any case, the data obtained in this study, along with results reported in the specialized literature, provided objective, replicable, and easy-to-use information for preparing the stimulus used in for newborns and breastfed infants. This information assists speech-language therapists and radiologists in addressing the challenge of creating examination conditions that closely mimic the infant’s feeding routine. Accurate results are crucial in order to avoid harmful indications, such as premature weaning or an incorrect diet consistency, which could put infants at risk for respiratory compromise, malnutrition, and dehydration. Therefore, accurate results are key to ensure their well-being, development, psychological and organic health.

The possible variability in the composition of breast milk, which was not observed in this research, and among other infant formulas available on the market does not allow us to take the study results as a general rule. Future studies should address statistical analysis with controlled variables, such as the time of day when the sample is collected, maternal conditions or establishing that the analysis occurs immediately after the sample has been collected. It will be also of interest to the scientific community to evaluate and determine the degree to which the small differences in viscosities do or do not impact the efficiency of swallowing especially considering infants^(18,21)^. Another consideration to be further explored in the future is to what extent the behavior of the fluid in the syringe mimics the behavior in contact with the oral and pharyngeal mucosa. The current research did not evaluate the swallowing performance of neonates in vivo, considering these differences, so the clinical impact cannot be accurately determined. It is also necessary to consider the wide variety of shear rates in the biomechanics of swallowing, in the different oral, pharyngeal, and esophageal phases^(29,30)^.

## CONCLUSION

The current research, comparing the behavior of liquids using two different methodologies (rheological evaluation and IDDSI flow test) made it possible to understand the behavior of liquids, frequently recommended in Brazil, for newborns and infants aged up to six months, added with liquid barium sulfate, using a specific thickener recommended for breast milk and newborns, in the specific and adequate dilution. The study revealed variability in viscosity among the formulations. While the addition of liquid barium does lead to increased viscosity, it does not follow a linear pattern, indicating that the products react differently. Therefore, it is essential for the clinician to be familiar with replicable methods of analyzing the viscosity of liquids to adequately prepare the stimuli to be offered during the examination, ensuring greater accuracy in evaluating swallowing dynamics and recommending a safe diet for the patient.

## Footnotes

1 mPa.s (millipoise) is a unit of dynamic viscosity, commonly used in the field of fluid mechanics and rheology. It measures how resistant a fluid is to flow under an applied force or stress. Millipoise is frequently used to measure the viscosity of liquids. Water at room temperature (about 20 °C) has a viscosity close to 0,1 millipoise , while substances like honey or motor oil have significantly higher viscosities.
